# Long-term results of three-part penile prosthesis implantation with Ectopic reservoir placement in the treatment of erectile dysfunction: is supramuscular tubulation a reliable method?

**DOI:** 10.1186/s12610-024-00225-2

**Published:** 2024-06-03

**Authors:** Yunus Erol Bozkurt, Caner Buğra Akdeniz, Bilali Habeş Gümüş

**Affiliations:** 1 Department of Urology, Manisa Merkez Efendi State Hospital, Manisa, Türkiye; 2 Department of Urology, İzmir Foça State Hospital, İzmir, Türkiye; 3 Faculty of Medicine, Department of Urology, Manisa Celal Bayar University, Manisa, Türkiye

**Keywords:** Penile prosthesis, Penile implantation, Erectile dysfunction, Prothèse pénienne, Implantation pénienne, Dysfonction érectile

## Abstract

**Background:**

Penile prosthesis implantation is the last resort for refractory erectile dysfunction. Reservoir placement is one of the biggest challenges in inflatable penile prosthesis implant surgery in several cases, especially in patients with abnormal pelvic anatomy. Ectopic reservoir placement with supramuscular approach has many advantages in these cases.

**Results:**

No complications were encountered except wound site infection in 2 patients which could be controlled with antibiotic treatment. EDITS scores were not statistically different between patients divided into 2-year groups according to follow-up time. Median values of EDITS scores were high in all groups, suggesting that couples had high sexual satisfaction both in the long term and in the short term.

**Conclusions:**

We recommend placement of the supramuscular tube and reservoir through the incision described, especially in patients whose pelvic anatomy has been modified by lower abdominal surgery.

**Supplementary Information:**

The online version contains supplementary material available at 10.1186/s12610-024-00225-2.

## Résumé

**Contexte:**

L’implantation d’une prothèse pénienne est le dernier recours pour les dysfonctions érectiles réfractaires. La mise en place d’un réservoir est l’un des plus grands défis de la chirurgie d’implant de prothèse pénienne gonflable dans de nombreux cas, en particulier chez les patientes présentant une anatomie pelvienne anormale.

**Résultats:**

Aucune complication n’a été rencontrée, à l’exception d’une infection du site de la plaie chez 2 patients, qui a pu être contrôlée par un traitement antibiotique. Les scores du questionnaire EDITS ne furent pas statistiquement différents entre les patients classés en groupes de 2 ans en fonction du temps de suivi. Les valeurs médianes des scores du questionnaire EDITS étaient élevées dans tous les groupes, ce qui suggère que les couples avaient une satisfaction sexuelle élevée à la fois à court et à long termes.

**Conclusions:**

Nous recommandons la mise en place du tube supramusculaire et du réservoir à travers l’incision décrite, en particulier chez les patients dont l’anatomie pelvienne a été antérieurement modifiée par une chirurgie abdominale basse.

## Introduction

Erectile dysfunction (ED) is an important health problem affecting men’s quality of life. The prevalence of erectile dysfunction in men aged ≥ 40 years in Turkey is 33%. The severity of erectile dysfunction was mild in 76.9%, moderate in 16.3% and severe in 5.7% of cases. Age, diabetes, hypertension, atherosclerosis, dyslipidemia and lower urinary tract disorders are the determining factors for the presence of erectile dysfunction [1]. Penile prosthesis implantation is an appropriate and permanent solution for patients who do not respond adequately to treatment despite the use of oral phosphodiesterase-5 inhibitors and intracavernosal vasoactive agents, are bothered by side effects or want a permanent solution to their problems [2]. Implantation of a 3-piece inflatable penile prosthesis (IPP) with a reservoir for fluid storage is associated with approximately 85–90% patient satisfaction and improved sexual quality of life. However, patients with Peyronie’s disease, radical prostatectomy, or body mass index > 30 kg/m2 have been shown to have a lower degree of increased satisfaction [3, 4].

Various modifications to IPP reservoirs since the 1970s have reduced the likelihood of reservoir-related mechanical failure in current models. Based on the location of the reservoirs, they can be divided into two groups: high submuscular (HSM) and traditional space of Retzius (SOR). Intestinal obstruction and herniation were observed in 2.3% of HSM reservoirs. Bladder erosion, vascular injury and reservoir herniation were seen in 4.6% of traditional SOR reservoir placements [5, 6]. The SOR reservoir is implanted blindly through either the penoscrotal or infrapubic incision. A complication during placement of the reservoir by an inexperienced surgeon can make a relatively simple procedure extremely difficult. In addition, surgical experience has a significant impact on the risk of prosthesis infection. Complications related to the post-operative reservoir lead to mechanical failure of the device and a decrease in patient satisfaction [7, 8]. Cases of HSM reservoir and IPP implantation in our clinic were retrospectively reviewed. The reservoir of the IPP and the tubing between them were implanted using a different method than the cases reported in the literature. We will discuss the effect of the method used in our study on patient satisfaction and postoperative complications.

## Material and method

The ethics committee of Manisa Celal Bayar University approved the study protocol with approval number 20478486-050.04.04. After the Ethics Committee approved the study, patients who underwent penile prosthesis implantation between 1 January 2014 and 31 December 2022 were retrospectively analysed. The reservoir and tubing of the 40 patients included in the study were implanted in the same way by a single surgeon.

Patients aged 18 years and older who had undergone IPP surgery and had a follow-up period of at least 1 year were included in the study. Patients with Peyronie’s disease, no deformity or penile curvature ≤ 30° were considered eligible for the study. Patients with preoperative penile curvature > 30° or severe penile fibrosis (moderate to severe penile deformity), patients with reservoir implanted in the SOR, and patients with penile prosthesis other than 3-piece implant were excluded.

**Fig. 1:**
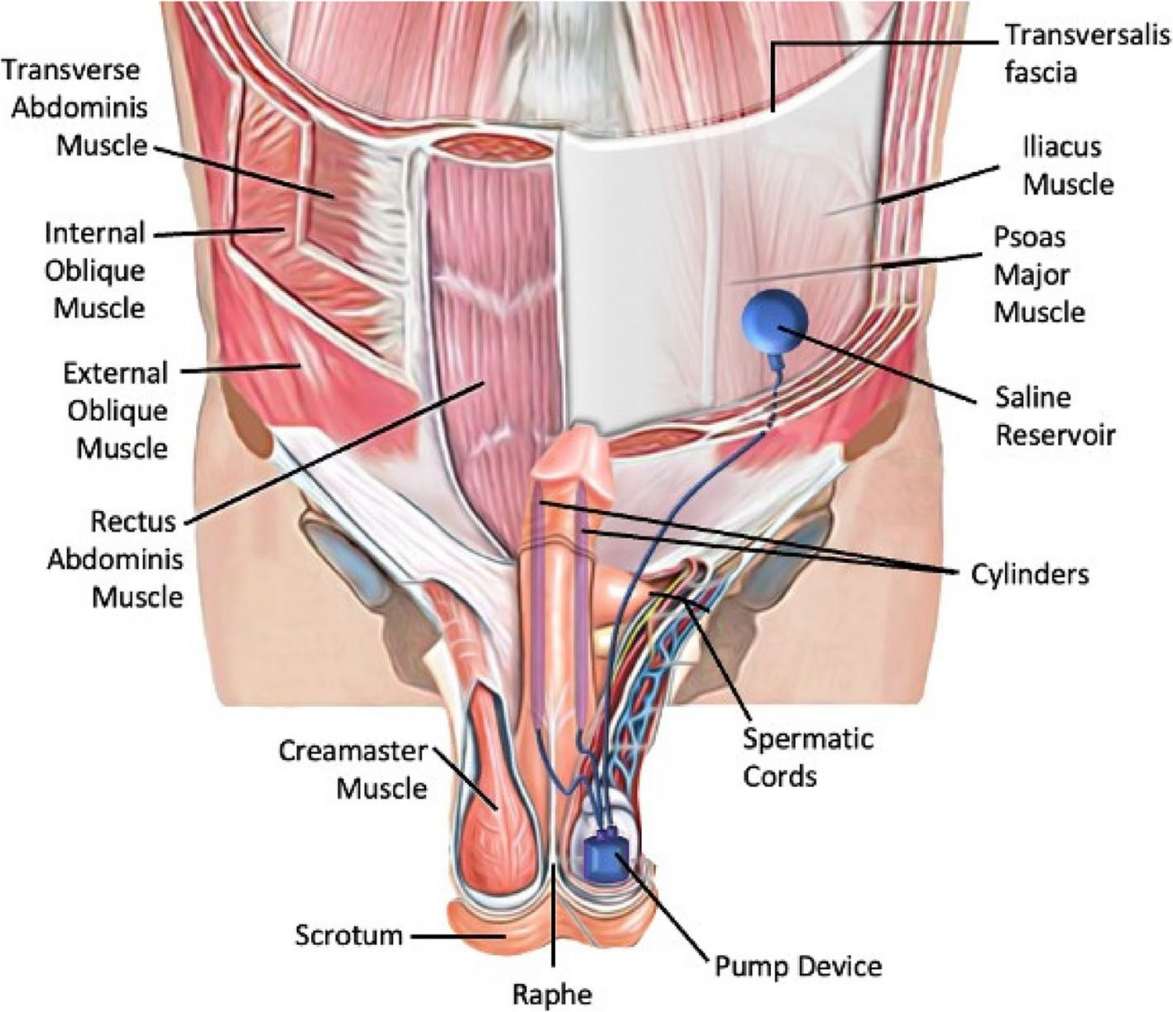
The original figure shows the placement of the 3-piece penile prosthesis and reservoir

**Fig. 2:**
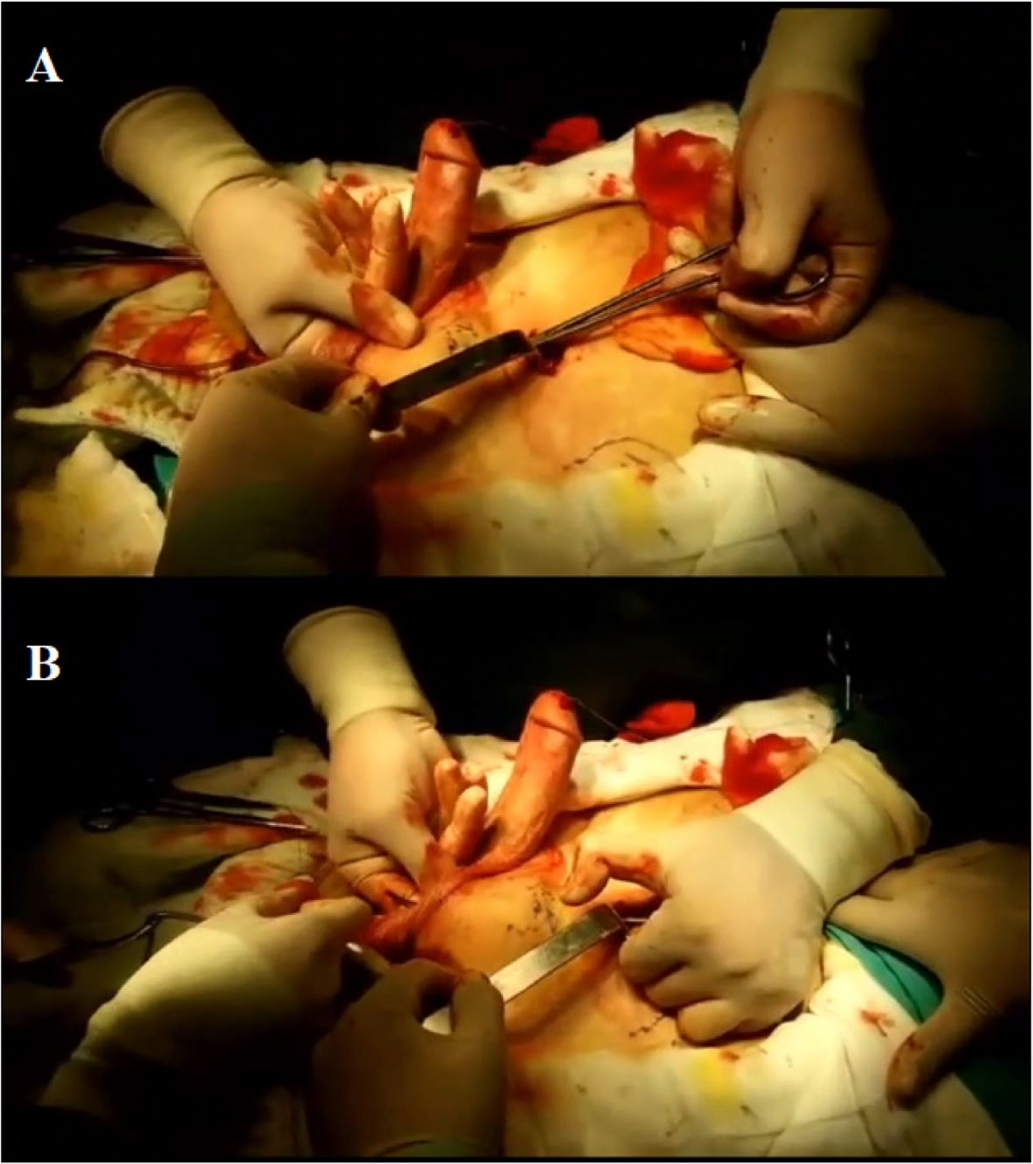
**A**: From the penoscrotal incision where the penile prosthesis is inserted, the tube to be connected to the reservoir is passed into the reservoir incision using a Kely clamp guided by the index finger. **B**: The tube is passed under the subcutaneous fat and over the fascia of the external abdominal muscle

The external oblique, internal oblique and transverse abdominal muscles were traversed by making a 3 cm transverse reservoir incision from 1/3 distal to the line between the umbilicus and the anterior superior iliac spine (Fig. 1). The reservoir space was created between the transverse abdominal muscle and the transverse fascia by bi-manual blunt dissection. Through the penoscrotal incision where the IPP was placed, the tube to be connected to the reservoir was directed to the reservoir incision with a Kely clamp guided by the index finger and moved between under the subcutaneous fat and over the fascia of the external abdominal muscle (Fig. 2). After the reservoir was placed through the reservoir incision and inflated, the fascia and muscles were properly sutured and closed.

**Fig. 3:**
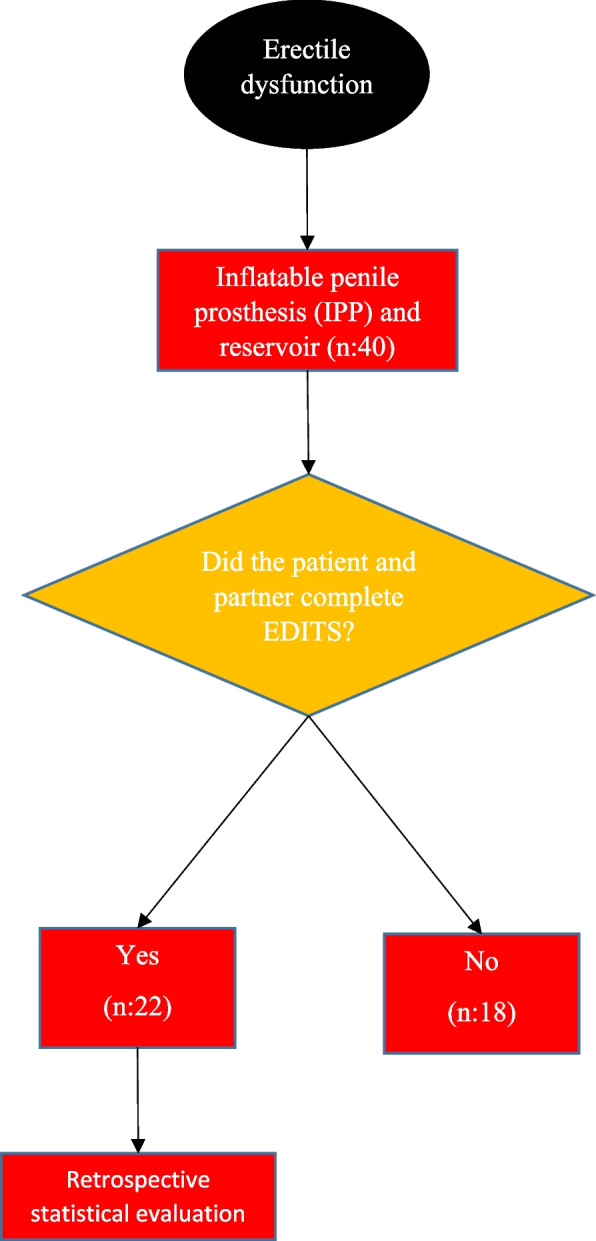
Flowchart of the study

When the patient files were analysed retrospectively, it was seen that 22 of the 40 patients who applied for control examination completed the Erectile Dysfunction Treatment Satisfaction Inventory (EDITS) questionnaire. The EDITS is a questionnaire designed to assess satisfaction with erectile dysfunction treatment and the effect of patient and partner satisfaction on treatment adherence. The questionnaire scores of 22 patients who completed the EDITS form were analysed by grouping them into postoperative years 1 and 2, years 3 and 4, and years 5 and 6 (Fig. 3). The patient version of EDITS consists of 11 questions and is scored from 0 to 4. The partner version of EDITS consists of 5 questions and is scored as the patient version. The average of the total score is calculated and multiplied by 25 and scored on a scale between 0 (least satisfied) and 100 (most satisfied) [9].

### Statistical analysis

Statistical analysis was performed using SPSS version 26 software. The numerical data of our study were not normally distributed according to the Kolmogorov-Smirnov test. The EDITS score data were divided into 3 groups according to the number of postoperative years, and the data were analysed using the Kruskal-Wallis test. Spearman’s correlation test was used to analyse the correlation between EDITS score data and patient age. *P* values < 0.05 were considered significant.

## Results

**Fig. 4:**
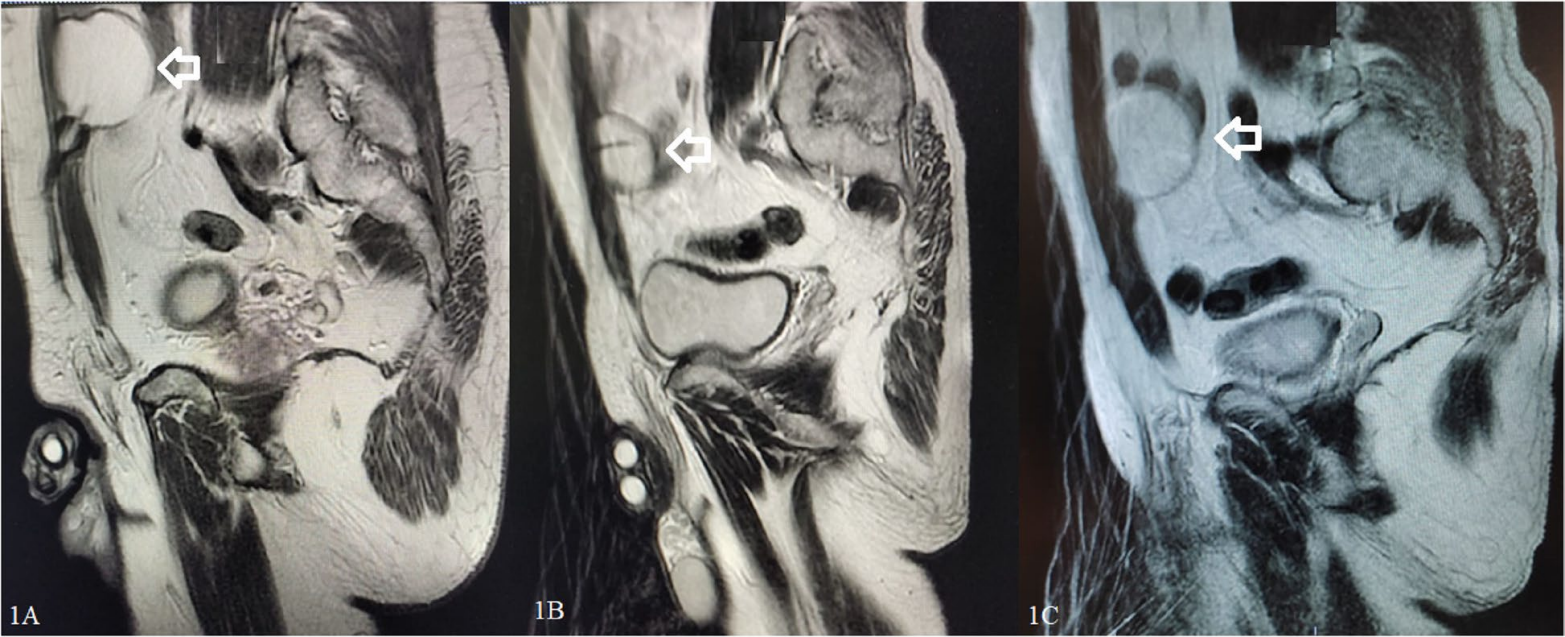
Abdominal magnetic resonance imaging of different patients at postoperative 2nd year (1A), 4th year (1B) and 6th year (1C). The arrows shows the location of the inflatable penile prosthesis reservoir in all patients

**Fig. 5:**
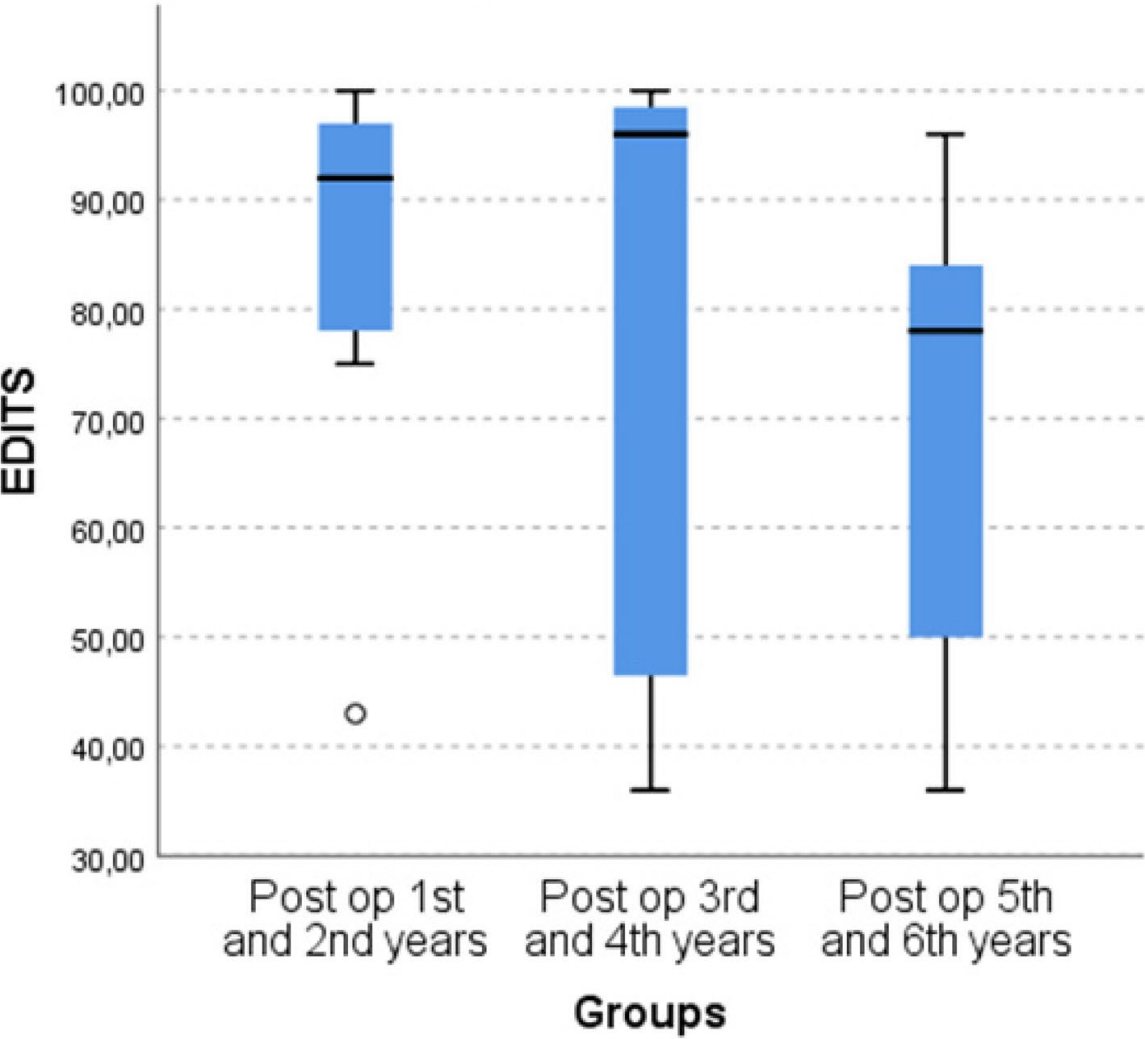
Distribution of EDITS scores of patients in the 1st and 2nd years (median score: 92, minimum score: 43 and maximum score: 100), 3rd and 4th years (median score: 96, minimum score: 36 and maximum score: 100) and 5th and 6th years (median score: 36, minimum score: 78 and maximum score: 96) post operatively according to groups

**Fig. 6:**
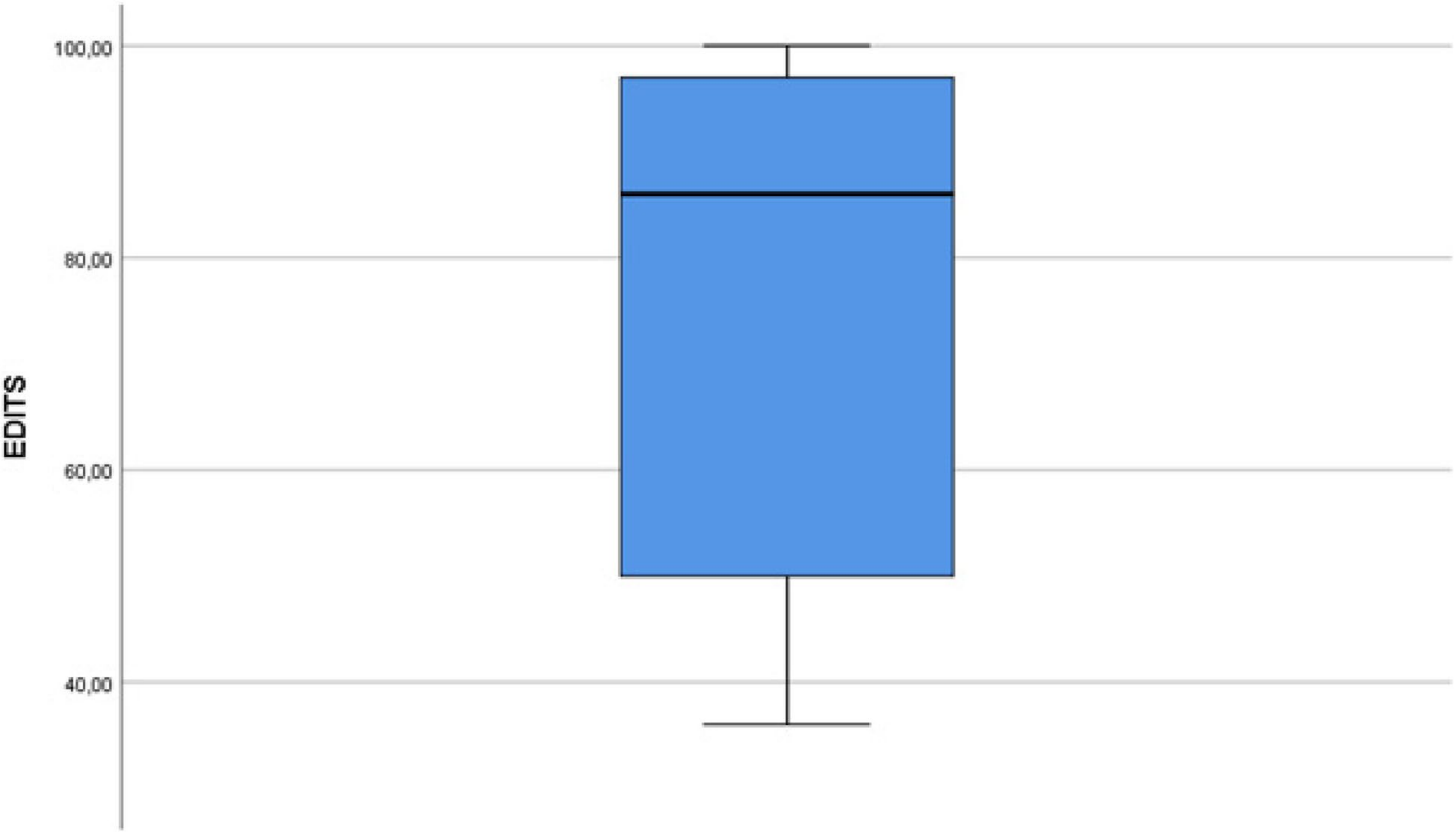
Distribution of EDITS scores. (median score: 86, minimum score: 36 and maximum score: 100). EDITS: Erectile Dysfunction Treatment Satisfaction Inventory

 The absence of reservoir migration in 40 patients was confirmed by ultrasound imaging during the control examination, and no negative results were observed in the working dynamics of the prosthesis due to the high localisation of the reservoir. No cases of bladder, bowel, blood vessel, spermatic cord, or nerve injury were reported. Moreover, not a single case of intravesical reservoir insertion, reservoir herniation, visibility, or palpability was recorded. 38 of 40 patients had no complications. Post operatively, 2 patient developed wound infection. However, it was taken under control with antibiotic treatment in about 1 week. Abdominal magnetic resonance imaging of 3 patients at postoperative years 2, 4 and 6 shows the location of the IPP reservoir (Fig. 4). Age, EDITS score, predisposing factors and follow-up data of 22 patients are shown in Table 1. The median score of the 22 patients who completed the EDITS questionnaire was 86, median follow-up was 3 years and the median age of these patients was 53 years. As a result of the Kruskal-Wallis test, no statistically significant relationship was found in terms of age groups (p: 0.4) (Fig. 5). This result shows that the postoperative satisfaction of patients and their partners is not affected by the time elapsed. The results of the EDITS score show a high level of postoperative satisfaction in most patients and their partners (Fig. 6).

**Table 1:** Age, EDITS score, predisposing factor and post follow-up time data of 22 patients

Patient	Age	EDTIS score	Postoperative Follow-up (year)	Predisposing Factor
1	53	75	1	Priapism
2	40	84	6	Spinal Injury
3	63	36	5	Radical Prostatectomy
4	45	88	2	Diabet
5	53	88	5	Penile Venous Leakage
6	59	97	4	Benign Prostatic Hyperplasia
7	49	43	3	Penile Venous Leakage
8	47	50	3	Diabet
9	56	97	1	Radiotherapy
10	65	78	1	Radical Prostatectomy
11	51	100	4	Diabet
12	57	96	2	Penile Venous Leakage
13	44	50	5	Diabet
14	69	36	3	Hypertension
15	45	100	2	Peyronie’s Disease Diabet
16	70	84	1	Penile Venous Leakage
17	66	97	1	Radical Prostatectomy
18	56	43	1	Radiotherapy
19	52	96	3	Diabetes Hypertension
20	48	96	5	Spinal Injury
21	56	100	3	Diabetes
22	42	100	2	Penile Venous Leakage

*EDITS* Erectile Dysfunction Treatment Satisfaction Inventory

According to Sperarman’s correlation test, no statistical correlation was found between patients’ age and their EDITS scores (p:0.4). This result shows that the postoperative satisfaction of the patient and their partner did not change with age. Spinal injury, radiotherapy, radical prostatectomy, priapism, penile venous leakage, diabetes and benign prostatic hyperplasia were found to be predisposing factors for erectile dysfunction.

## Discussion

In this study, we reported the results of the 3-piece IPP reservoir, which was implanted using a method different from that described in the literature. None of the 40 patients had a complication related to the reservoir. The majority of the 22 patients and their partners who completed the EDITS form were satisfied with the IPP implantation.

The traditional site for IPP reservoir implantation was the SOR via blunt perforation through the transversalis fascia or direct incision in the midline suprapubic portion of the rectus fascia. With the development of IPP reservoirs and closure valves, surgeons have chosen HSM as an alternative to SOR. The aim is to avoid vascular and intestinal complications [10].

Preferring HSM reservoir placement in patients with difficulties in prosthetic surgery such as robotic radical prostatectomy, inguinal and pelvic fibrosis, radical cystectomy and inguinal hernia repair would be more accurate in terms of postoperative complications [11].

As a significant number of patients with IPP implant had these difficulties, we preferred HSM reservoir and supramuscular tube for all patients to avoid complications in patients with SOR implanted reservoirs. In the study by Ziegelmann et al., bilateral IPP reservoir implantation was performed in the HSM cavity of 10 male cadavers. In order to determine the exact anatomical position of the reservoir after the procedure, the abdominal cavities of the cadavers were opened and the abdominal walls were fragmented. Only 35% were identified at the intended location of the HSM. Others were located between the external oblique fascia and the internal oblique fascia (45%), the retroperitoneal layer (10%), the preperitoneal layer (5%) and the intraperitoneal space (5%) [12]. The supramuscular tube and reservoir incision method provided a comfortable field of view of the transverse fascia for reservoir implantation. Data from the cadaver and simulation labs, attended by 31 urology registrars, showed that 42% of participants were reluctant to place the reservoir in the Retzius space or a submuscular area. However, survey data at the end of the course showed that 90% of participants gained confidence in reservoir placement [13].

Although these data cannot be generalised to the entire urologist population, it is reasonable to assume that the data is representative of many young urologists. Considering that penile prosthesis implantation is not always performed in high-volume clinics, reservoir implantation with supramuscular tube and reservoir incision is a non-blinded, relatively simple and low-risk method.

Prior to the advent of robotic-assisted radical prostatectomy (RARP), SOR was preferred because it was easily accessible, the reservoir was not palpable and it provided a low pressure environment. With advancing technology, the number of RARPs is increasing and is becoming more common than open prostatectomy [14]. During RARP, the SOR is exposed to the peritoneal cavity as the peritoneum covering the pelvis and bladder is resected. It is unpredictable whether this exposed area will be peritonised again. Because the transverse fascia is dissected during conventional IPP implantation, the risk of the reservoir entering the peritoneum is increased [15]. We believe that this risk is avoided with the alternative reservoir placement technique described above.

Alternative methods of reservoir implantation have been described by many authors. According to a study performed with IPP by Bruce B. Garber et al., the IPP reservoir was placed subcutaneously in 8 obese patients and it was reported that 7 patients recovered without any problems [16]. In another alternative method, Doron S. Stember et al. performed a cylindrical reservoir implant between the transverse fascia and the peritoneum. Palpation and herniation of the reservoir were observed at relatively low levels in these patients [17]. Mykoniatis et al. described a modified SOR reservoir implantation in their study. The method of perforating the external oblique fascia, placing the reservoir and closing the fascia with sutures was used in 253 patients and no complications were noted [18]. However, increasing the number of patients will make the data more consistent.

### Limitations of the study

The limitations of our study are that it is retrospective and EDITS scoring was obtained from a small patient population.

## Conclusion

HSM placement of an IPP reservoir is a relatively simple technique that can be used to avoid the potential catastrophic complications associated with traditional retroperitoneal reservoir placement. We recommend that the supramuscular tube and reservoir be placed through the incision we have described, particularly in patients whose pelvic anatomy has been altered by lower abdominal surgery. We do not recommend that the method we have described replaces all other methods, but it should be kept in mind as an alternative and easily applicable option for prosthetic reservoir implantation.

### Supplementary Information

**Supplementary Material 1.**

## Data Availability

The data is avaiable at a reasonable request to the corresponding authors.

## Abbreviations

EDITS: Erectile Dysfunction Treatment Satisfaction Inventory
ED: Erectile dysfunction
HSM: High submuscular
IPP: Inflatable penile prosthesis
RARP: Robotic-assisted radical prostatectomy
SOR: Space of Retzius
