# Supplementary material for: Long-term results of three-part penile prosthesis implantation with Ectopic reservoir placement in the treatment of erectile dysfunction: is supramuscular tubulation a reliable method?
Source: Basic Clin Androl. 2024 Jun 3;34:8. doi: 10.1186/s12610-024-00225-2 (PMC11149340; doi:10.1186/s12610-024-00225-2)
Supplement: Supplementary file 1 — Supplementary Material 1. [file 12610_2024_225_MOESM1_ESM.pdf]

APPENDIX I. THE EDITS: ERECTILE DYSFUNCTION INVENTORY OF TREATMENT SATISFACTION, PATIENT VERSION

Stanley E. Althof, Ph.D. & Eric W. Corty, Ph.D.

Name or ID number: __________

Date: ______

What treatment method are you currently using? __________

The questions in this inventory ask about a sensitive topic, your sexual life with your wife or partner as well as your attitude toward and expectations from the treatment method you are using to help with your erection problem. Please an- swer the questions as honestly and candidly as you can. If any questions or terms are unclear, please ask for clarification.

1. Overall, how satisfied are you with this treatment?
   a. Very satisfied
   b. Somewhat satisfied
   c. Neither satisfied nor dissatisfied
   d. Somewhat dissatisfied
   e. Very dissatisfied
2. During the past four weeks, to what degree has the treatment met your expectations?
   a. Completely
   b. Considerably
   c. Half way
   d. A little
   e. Not at all
3. How likely are you to continue using this treatment?
   a. Very likely
   b. Moderately likely
   c. Neither likely nor unlikely
   d. Moderately unlikely
   e. Very unlikely
4. During the past four weeks, how easy was it for you to use this treatment?
   a. Very easy
   b. Moderately easy
   c. Neither easy nor difficult
   d. Moderately difficult
   e. Very difficult
5. During the past four weeks, how satisfied have you been with how quickly the treatment works?
   a. Very satisfied
   b. Somewhat satisfied
   c. Neither satisfied nor dissatisfied
   d. Somewhat dissatisfied
   e. Very dissatisfied
6. During the past four weeks, how satisfied have you been with how long the treatment lasts?
   a. Very satisfied
   b. Somewhat satisfied
   c. Neither satisfied nor dissatisfied
   d. Somewhat dissatisfied
   e. Very dissatisfied
7. How confident has this treatment made you feel about your ability to engage in sexual activity?
   a. Very confident
   b. Somewhat confident
   c. It has had no impact
   d. Somewhat less confident
   e. Very much less confident
8. Overall, how satisfied do you believe your partner is with the effects of this treatment?
   a. Very satisfied
   b. Somewhat satisfied
   c. Neither satisfied nor dissatisfied
   d. Somewhat dissatisfied
   e. Very dissatisfied
9. How does your partner feel about your continuing to use this treatment?
   a. My partner absolutely wants me to continue
   b. My partner generally prefers me to continue
   c. My partner has no opinion
   d. My partner generally prefers me to stop
   e. My partner absolutely wants me to stop
10. How natural did the process of achieving an erection feel when you used this treatment over the past four weeks?
    a. Very natural
    b. Somewhat natural
    c. Neither natural nor unnatural
    d. Somewhat unnatural
    e. Very unnatural
11. Compared to *before you had an erection problem* how would you rate the naturalness of your erection when you used this treatment over the past four weeks in terms of hardness?
    a. A lot harder than before I had an erection problem
    b. Somewhat harder than before I had an erection prob- lem
    c. The same hardness as before I had an erection problem
    d. Somewhat less hard than before I had an erection problem
    e. A lot less hard than before I had an erection problem

Thank you for having completed the questionnaire.

APPENDIX II. THE EDITS: ERECTILE DYSFUNCTION INVENTORY OF TREATMENT SATISFACTION, PARTNER VERSION

Stanley E. Althof, Ph.D. & Eric W. Corty, Ph.D.

Name or ID number: __________

Date: ______

What treatment method is your husband or partner currently using for his erection problem? __________

The questions in this inventory ask about a sensitive topic, your sexual life with your husband or partner as well as your attitudes and experiences regarding treatment for his erection problem. Please answer the questions as honestly and candidly as you can. If any questions or terms are unclear, please ask for clarification.

1. Overall, how satisfied are you with this treatment for your husband's or partner's erection problem?
   a. Very satisfied
   b. Somewhat satisfied
   c. Neutral; neither satisfied nor dissatisfied
   d. Somewhat dissatisfied
   e. Very dissatisfied
2. During the past four weeks, to what degree has the treatment met your expectations?
   a. Completely
   b. Considerably
   c. Half way
   d. Somewhat
   e. Not at all
3. Over the past four weeks, how has this treatment affected your sense of being sexually desirable?
   a. It has made me feel much more sexually desirable
   b. It has made me feel somewhat more sexually desirable
   c. It has had no impact on my sense of being sexually desirable
   d. It has made me feel somewhat less sexually desirable
   e. It has made me feel less sexually desirable
4. Over the past four weeks, how satisfied have you been with how long this treatment enhances your husband's or partner's ability to achieve an erection?
   a. Very satisfied
   b. Somewhat satisfied
   c. Neutral, neither satisfied nor dissatisfied
   d. Somewhat dissatisfied
   e. Very dissatisfied
5. How do you think your husband or partner feels about continuing this treatment?
   a. I think that he very much wants to continue using this treatment
   b. I think that he somewhat wants to continue using this treatment
   c. I think my partner feels neutral about continuing to use this treatment
   d. I think that he somewhat wants to discontinue using this treatment
   e. I think that he very much wants to discontinue using this treatment

Thank you for having completed the questionnaire.
